# Characterization of the Mantle Transcriptome of Yesso Scallop (*Patinopecten yessoensis*): Identification of Genes Potentially Involved in Biomineralization and Pigmentation

**DOI:** 10.1371/journal.pone.0122967

**Published:** 2015-04-09

**Authors:** Xiujun Sun, Aiguo Yang, Biao Wu, Liqing Zhou, Zhihong Liu

**Affiliations:** Yellow Sea Fisheries Research Institute, Chinese Academy of Fishery Sciences, Qingdao, China; University of Lausanne, SWITZERLAND

## Abstract

The Yesso scallop *Patinopecten yessoensis* is an economically important marine bivalve species in aquaculture and fishery in Asian countries. However, limited genomic resources are available for this scallop, which hampers investigations into molecular mechanisms underlying their unique biological characteristics, such as shell formation and pigmentation. Mantle is the special tissue of *P*. *yessoensis* that secretes biomineralization proteins inducing shell deposition as well as pigmentation on the shells. However, a current deficiency of transcriptome information limits insight into mechanisms of shell formation and pigmentation in this species. In this study, the transcriptome of the mantle of *P*. *yessoensis* was deeply sequenced and characterized using Illumina RNA-seq technology. A total of 86,521 unique transcripts are assembled from 55,884,122 reads that passed quality filters, and annotated, using Gene Ontology classification. A total of 259 pathways are identified in the mantle transcriptome, including the calcium signaling and melanogenesis pathways. A total of 237 unigenes that are homologous to 102 reported biomineralization genes are identified, and 121 unigenes that are homologous to 93 known proteins related to melanin biosynthesis are found. Twenty-three annotated unigenes, which are mainly homologous to calmodulin and related proteins, Ca2+/calmodulin-dependent protein kinase, adenylate/guanylate cyclase, and tyrosinase family are potentially involved in both biomineralization and melanin biosynthesis. It is suggested that these genes are probably not limited in function to induce shell deposition by calcium metabolism, but may also be involved in pigmentation of the shells of the scallop. This potentially supports the idea that there might be a link between calcium metabolism and melanin biosynthesis, which was previously found in vertebrates. The findings presented here will notably advance the understanding of the sophisticated processes of shell formation as well as shell pigmentation in *P*. *yessoensis* and other bivalve species, and also provide new evidence on gene expression for the understanding of pigmentation and biomineralization not only in invertebrates but also probably in vertebrates.

## Introduction

Biomineralization, the processes by which organisms form minerals, occurs in a majority of metazoan taxa, such as vertebrates, corals and molluscs. The well-known biominerals, such as bone and mollusc shell, are complex composites of inorganic minerals and organic macromolecules, exhibiting unusual toughness, strength and hardness [1, 2]. An understanding of the basis of biomineralization is of widespread importance in biology, ecology and engineering. The shells of molluscs are distinctive and often beautiful structures that are attracting growing research interest from many different aspects, such as research on shell formation and pigmentation, paleontological study of shell fossils, biomaterial study on pearl formation, and ecological and environmental studies regarding the impact of ocean acidification on shelled molluscs [3–7].

Mollusc shells are natural biomaterials, composed mainly of calcium carbonate (~95%) and a small amount of organic macromolecules, including proteins, glycoproteins, polysaccharides and lipids [1, 8]. The combination of calcium carbonate crystals in either calcite or aragonite polymorphs with an organic matrix, enhances the mechanical properties of the shell, providing highly rigid protection of soft tissues from predators [9]. The organic macromolecules in mollusc shells are thought to play key roles in nucleation, orientation, morphology, polymorphism and organization of calcium carbonate crystallites of the shell [10]. For instance, two proteins involved in biomineralization, calmodulin and calmodulin-like protein, mediate many important signaling pathways by regulating crucial processes such as calcium transport and secretion, cell proliferation and differentiation, nucleation of aragonite, and regulation of calcite growth [11–13]. Chitin, synthesized via a complex transmembrane chitin synthase, is also a key component in mollusc shell formation, playing a functional role in controlling mineral deposition in mollusc shells [14, 15]. Perlucin may promote the nucleation and the growth of calcium carbonate crystals by increasing the precipitation of calcium carbonate and serving to connect the chitin and aragonite layers [16].

Most of organic matrix proteins in mollusc shells, including those mentioned above, are specifically secreted by the mantle epithelium. The anterior edge of the mantle tissue directs the formation of different structural layers of the shell, and controls the patterning of architectural and color features [17]. The matrix proteins responsible for shell formation are involved in assembly of the matrix, mineral phase, nucleation of aragonite tablets, and growth of the tablets and are a new focus of research for understanding the mechanisms of nacre deposition [18]. Aside from architectural features, the shells of many species of seashells, such as gastropods, oysters and scallops, have striking colors and color-patterns, with some shells containing pigments that are incorporated into the structure [17, 19, 20]. Melanins, which are probably formed in the secretory cells of the mantle edge, are the essential compound in these shell pigments [21]. The melanin biosynthetic pathway is regulated by tyrosinase, which is one of the phenoloxidases in melanogenesis [22, 23]. As indicated, molluscan tyrosinases are secreted from the mantle and transported to the prismatic shell layer, where they contribute to melanin biosynthesis and shell pigmentation [19, 24, 25].

Identification and characterization of genes involved in biomineralization and melanin biosynthesis are critically important for understanding mechanisms of shell formation and pigmentation in molluscs. However, few studies have conducted on this issue. Although the structure and function of many shell matrix proteins have recently been characterized, the vast majority of genes that govern the processes of biomineralization and melanogenesis remain largely unknown.

The transcriptome is the complete set of transcripts in a cell, which provides important information on gene expression and regulation for understanding the functional elements of the genome and molecular constituents of cells and tissues [26]. RNA-Seq, a high-throughput method for transcriptome sequencing, has been widely applied to model and non-model species [27, 28] and is an effective way to identify potential genes or pathways involved in specific biological processes. Significant progress has been made in understanding the processes of biomineralization in molluscs by RNA-Seq over the last few years, revealing an unprecedented, global view of the mantle transcriptome and numerous potential genes related to biomineralization [29–32].

**Fig 1:**
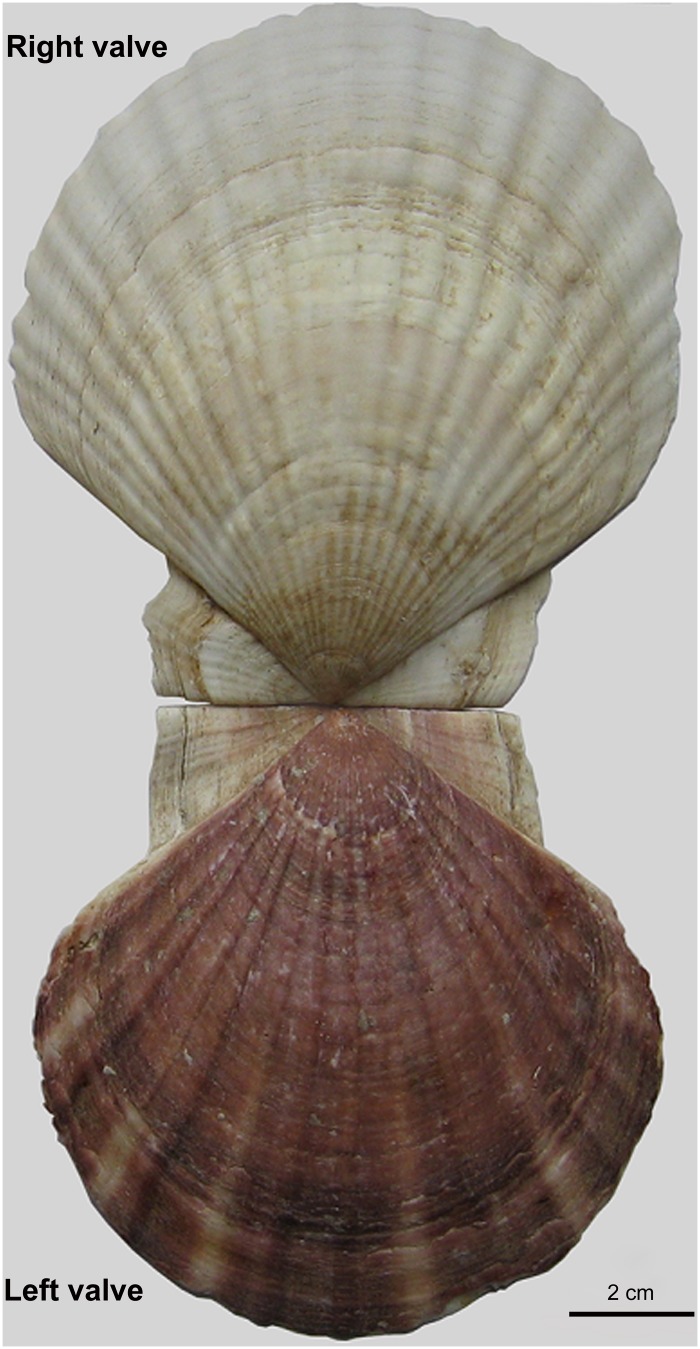
The shell picture for Yesso scallop *Patinopecten yessoensis*. The predominant color of the left valve is dark brown, while the absence of pigmentation occurs on the right valve.

The Yesso scallop *Patinopecten yessoensis* is a cold water bivalve that is naturally distributed along the coastline of northern Japan, the northern Korean Peninsula, and the Russian Far East [33, 34]. Both valves have smooth exterior surfaces, and are convex in the center. The predominant color of the left valve is dark brown, while the right valve lacks pigmentation (Fig 1). Due to its large and edible adductor muscle, *P*. *yessoensis* has become one of the most important maricultural species in northern China, since its introduction in 1982. Artificial production of Yesso scallop seed is of great importance for the aquaculture industry in hatcheries in China [34]. Previous studies on *P*. *yessoensis* have investigated aspects of artificial seed production and culture [35], polyploidy induction [36], interspecific hybridization [37], and population genetics [38]. Although transcriptome sequencing was performed for *P*. *yessoensis*, using a mix of diverse developmental stages and adult tissues [39], no transcriptome studies have been done to shed light on mechanisms of shell formation and pigmentations in this species. In the present study, we report the first assembly of a mantle transcriptome for *P*. *yessoensis* from Illumina-based RNA-Seq technology. The characterization of mantle transcriptome not only provides a global gene expression profile for the mantle of *P*. *yessoensis* but also allows identification of genes potentially involved in the process of shell formation and pigmentation. Furthermore, because of unique biological characteristics, such as shell shape polymorphisms and pigmentation in *P*. *yessoensis*, compared to the pearl oyster *Pinctada* traditionally used in biomineralization studies [31, 40], the exploration of shell formation in *P*. *yessoensis* could enrich theoretical insight into the biomineralization process, assist our understanding of the sophisticated processes of shell formation and pigmentation in this species, and also provide useful information for its culture.

## Methods

### Ethics Statement

The scallops used in the current study were marine-cultured animals, and all the experiments on scallops were conducted following institutional and national guidelines. No endangered or protected species was involved in the experiments of this study. No specific permission was required for the location of the culture experiment.

### Sample Collection and Preparation

Healthy *P*. *yessoensis* (mean shell length: 9.55 ± 0.42 cm; two-year-old adults) were obtained from a commercial hatchery in Yantai, China. The scallops were cultured in sand-filtered sea water at 14 ± 2°C with the salinity of 32 ± 2 psu for two weeks before processing. The culture experiment was carried out at our research base for shellfish genetic breeding in Jiaonan, China (119.97N; 35.88E). Scallops were fed with *Isochrysis galbana* and two-thirds of the culture water was exchanged every day. The mantle edge for both of left and right valves of six scallops was dissected and stored in RNAlater (Ambion) for RNA extraction. Total RNA was isolated with Trizol Reagent (Invitrogen). RNA purity was checked using the NanoPhotomete spectrophotometer (Implen, CA, USA) and the degradation and contamination of RNA was monitored on 1% agarose gels. The concentration of RNA was measured using Qubit RNA Assay Kit in Qubit 2.0 Flurometer (Life Technologies, CA, USA). RNA integrity was assessed using the RNA Nano 6000 Assay Kit for the Agilent Bioanalyzer 2100 system (Agilent Technologies, CA, USA). The RIN (RNA integrity number) values for all the RNA samples were ranging from 7.6 to 8.3.

### Library Preparation for Transcriptome Sequencing

A total amount of 3 μg RNA per individual was used as input material for RNA sample preparations. RNA samples from the six individuals were pooled in equal amounts to generate a mixed sample for library construction. The sequencing library was prepared according to the previous study [28], using NEBNext RNA Library Prep Kit for Illumina (NEB, USA), following manufacturer’s instructions. PCR products were purified (AMPure XP system) and library quality was assessed on the Agilent Bioanalyzer 2100 system. One library was generated and used for transcriptome sequencing in a single lane of Illumina HiSeq 2000 platform.

### Illumina Sequencing, Assembly, and Annotation

Paired-end sequencing of the library was done on an Illumina HiSeq 2000 platform for 100 cycles (Novogene Bioinformatics Technology Co. Ltd). Raw data was processed, first, with in-house Perl scripts to remove reads containing adapters or ambiguous nucleotides (if the proportion of “Ns” exceeded 10%) and reads of low quality (quality score of less than 5). After filtering, the clean reads were obtained and assembled into contigs using Trinity [41] as described for de novo transcriptome assembly without a reference genome (min_kmer_cov set to 2 and all other parameters set to default). Meanwhile, Q20, Q30 and GC-content of the data were calculated and used to filter the data for the downstream analyses.

The contigs were connected to generate scaffolds. Paired-end reads were used for the gap filling of scaffolds to get sequences that could not be extended on either end. These sequences were defined as transcripts, which were submitted to BLAST searches with annotation against the Nr (NCBI non-redundant protein sequences) and Nt (non-redundant nucleotide sequences) to cluster transcripts into clusters according to sequence similarities. The longest transcript in a clustering unit was selected as unigene. After sequence assembly, the unigene sequences were also aligned by BLASTX to protein databases such as PFAM (Protein family), Swiss-Prot, KEGG (Kyoto Encyclopedia of Genes and Genomes) and KOG (euKaryotic Ortholog Groups), with similarity cutoff of 30%, in order to retrieve proteins with the highest similarity, along with putative functional annotations. Annotation was prioritized by database (from highest to lowest priority, Nr, Nt, KEGG, Swiss-Prot, PFAM, GO and KOG). To obtain significant annotations, alignments with Nr, Nt and Swiss-Prot databases were carried out with a cut-off E-value of 10^-5^, while KOG and KEGG classification were done with a cut-off E-value of 10^-3^.

### Identification of Genes Involved in Biomineralization and Melanin Biosynthesis

Identification of biomineralization-related proteins was carried out, first, by searching key words of reported expressed genes in the mantle of molluscs, such as calmodulin, calmodulin-like protein, chitin synthase, perlucin and etc., in the BLASTX alignment results with Nr, Nt, KEGG, Swiss-Prot, PFAM, GO and KOG databases. Secondly, to identify transcripts encoding proteins potentially relevant to biomineralization, several methods were used to make the selections and predictions. The translated amino acid sequences of all transcripts were used to screen the signal peptides using SignalP 4.1 Server (http://www.cbs.dtu.dk/services/SignalP/) [42]. The obtained signal peptides were used to predict and filter for transmembrane domains using TMHMM Server 2.0 (http://www.cbs.dtu.dk/services/TMHMM/) [43]. Glycosylphosphatidylinositol (GPI) anchored proteins were detected using the GPI Prediction Server (http://mendel.imp.ac.at/sat/gpi/gpi_server.html) and then removed from the results [44, 45]. Predicting subcellular localization of proteins was performed to remove proteins targeted for mitochondria, chloroplast or other organelles, using TargetP v1.1 (http://www.cbs.dtu.dk/services/TargetP/) [46]. Since several biomineral-related proteins are known to have either repetitive motifs or domains of low complexity [4, 47], we identified proteins with tandem repeat units using XSTREAM (http://jimcooperlab.mcdb.ucsb.edu/xstream/) [48].

Because the important step of melanin biosynthetic pathway for both eumelanins and pheomelanins is catalysed by tyrosinase, the pathways of melanogenesis and tyrosine metabolism found in the KEGG database are considered as the most important metabolic pathways for melanin biosynthesis. All unigenes involved in these two pathways were selected and identified as genes potentially related to melanin biosynthesis in *P*. *yessoensis*. Meanwhile, transcripts related to pigment biosynthesis were screening BLASTX annotations for the key word “melanogenesis” or “melanin”. The unigene with maximum E-value was selected as the representative, when several unique transcripts were assigned to the same reference gene.

## Results

### Sequence Analysis and De Novo Assembly

Illumina sequencing generated 57.5 million raw reads (Table 1). After filtering, a total of 55.9 million reads remained, comprising 5.58 trillion nucleotides. The filtered reads used in this study have been deposited in the NCBI SRA database (accession number: SRP046039). The values of the Q20 percentage and Q30 percentage are 97.77% and 93.04%, respectively, and the error rate is 0.03%. The percentage of GC content for the clean reads is 41.49%.

**Table 1:** Statistics of Illumina sequencing in the mantle transcriptome of *Patinopecten yessoensis*.

Total raw reads	57,533,844
Total clean reads	55,884,122
Total clean bases (nt)	5,580,000,000
Q20 (%)	97.77
Q30 (%)	93.04
Error (%)	0.03
GC content (%)	41.49

**Fig 2:**
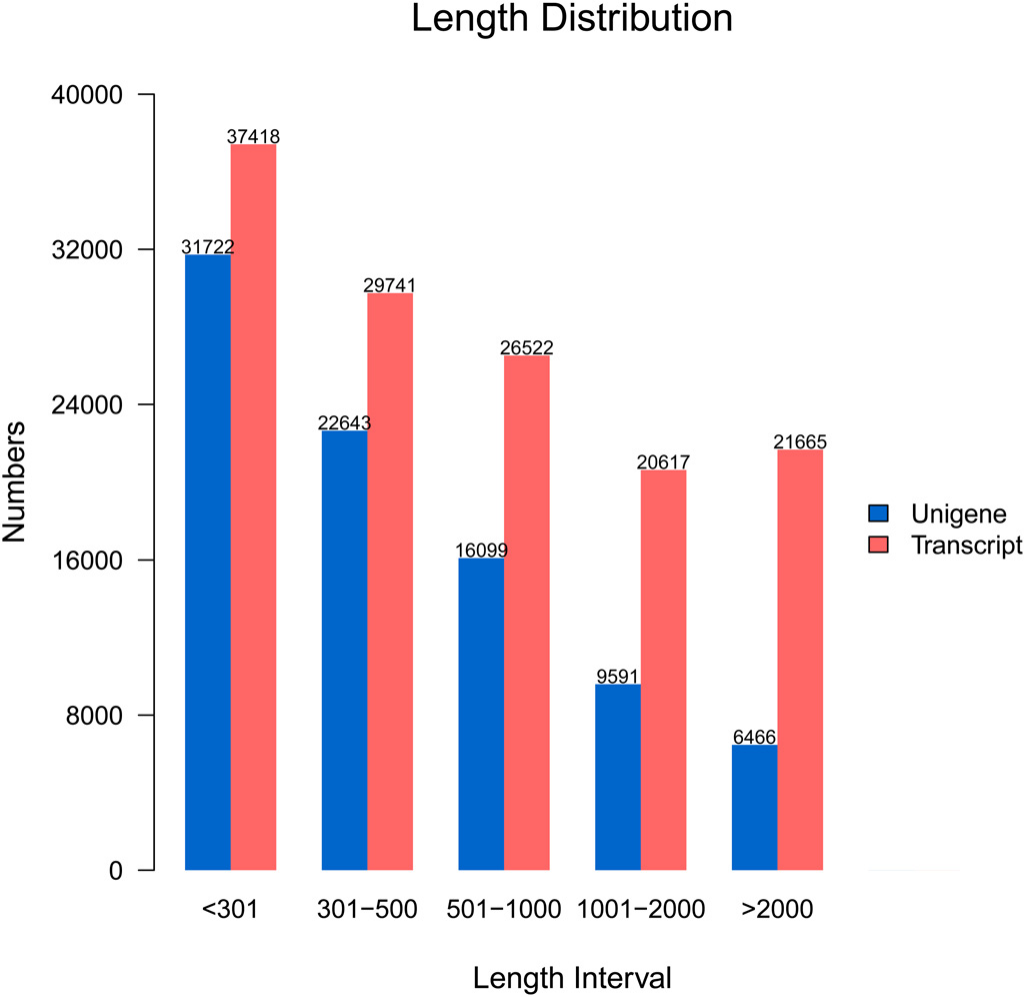
Sequence length distribution of transcripts and unigenes assembled from Illumina reads for the mantle transcriptome of *P*. *yessoensis*. The x-axis indicates length interval of transcripts and unigenes, and the y-axis indicates number of transcripts and unigenes for each size.

Filtered reads are assembled into 135,963 transcripts, with a total length of 152 million nucleotides (Table 2). The mean length of transcripts is 1,120 nucleotides, with N50 of 2,296. N50 length is defined as the contig length L for which 50% of all bases in the sequences are in contigs of length less than L. These transcripts are subsequently assembled into 86,521 unigenes, with the average length of 733 nucleotides, ranging from 201 to 32,355 nucleotides. To sum up, the total length of all unigenes is 63.4 million nucleotides or 41.6% of the length of all transcripts. The length-frequency distribution for unigenes and transcripts (Fig 2) shows declines both in the number of transcripts and in the ratio of unigenes to transcripts with increasing length. Most short reads (< 501 nt) are assembled into unigenes, while less than one-third of long reads (> 2000 nt) are assembled into unigenes, indicating that short reads are more likely to be assembled into unigenes.

**Table 2:** Statistics of de novo assembly for the mantle transcriptome of *P*. *yessoensis*.

Items	Total number	Total length (nt)	Mean length (nt)	N50
Transcripts	135,963	152,287,276	1,120	2,296
Unigenes	86,521	63,389,943	733	1,266

### Functional Annotation of Unigenes

**Fig 3:**
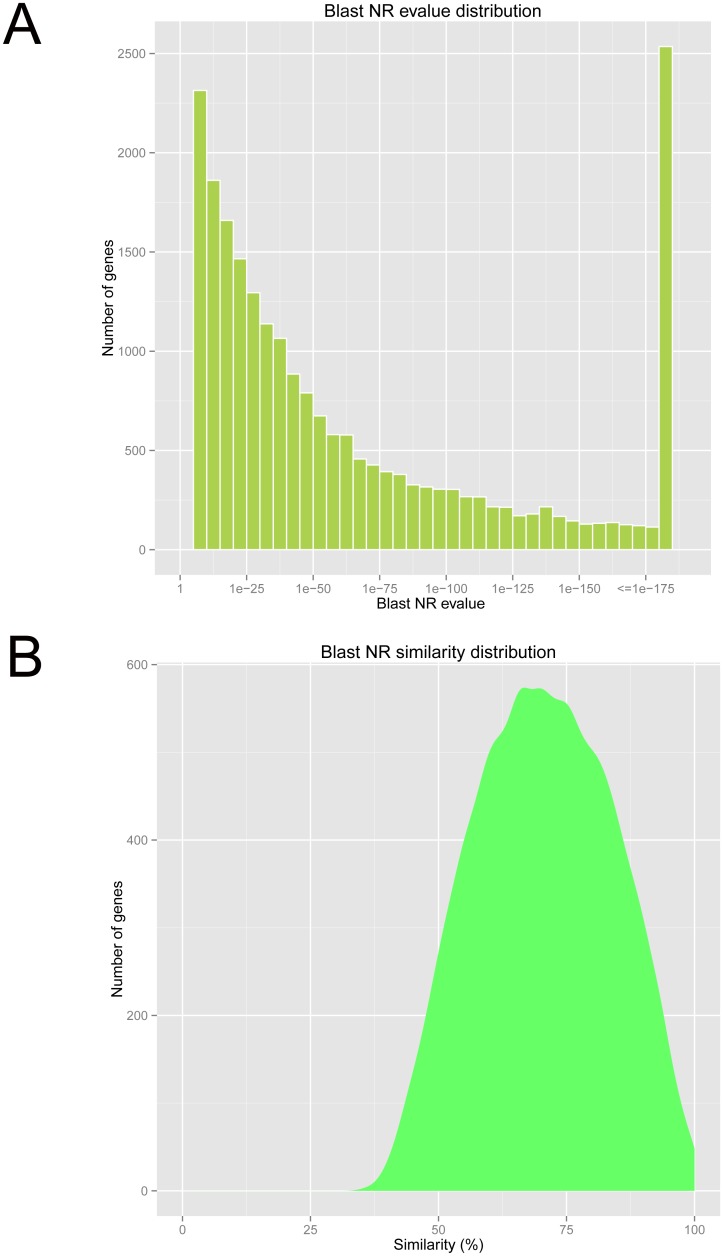
E-value and similarity distribution of unigenes with annotation to Nr (NCBI non-redundant protein sequences) database. (A) E-value distribution of annotated unigenes; (B) Similarity distribution of annotated unigenes.

Of the 86,521 unigenes, 28,228 (32.62%) are annotated by BLAST matches to at least one database. The highest percentage of unigenes is annotated in the Nr database, accounting for 25.83% of all unigenes, followed by 23.80% annotated in the GO database 23.45% in the PFAM database, and only 2.8% in the Nt database. Among unigenes successfully annotated in the Nr database, strong homology (E-value less than 10^-25^) is observed in 15,051 unigenes (67.34%; Fig 3A). There are 16,680 unigenes aligned with a similarity index higher than 60%, according to the similarity distribution (Fig 3B). More than 65% of the annotated unigenes (14,773) are homologous to the proteins from the Pacific oyster *Crassostrea gigas*.

**Fig 4:**
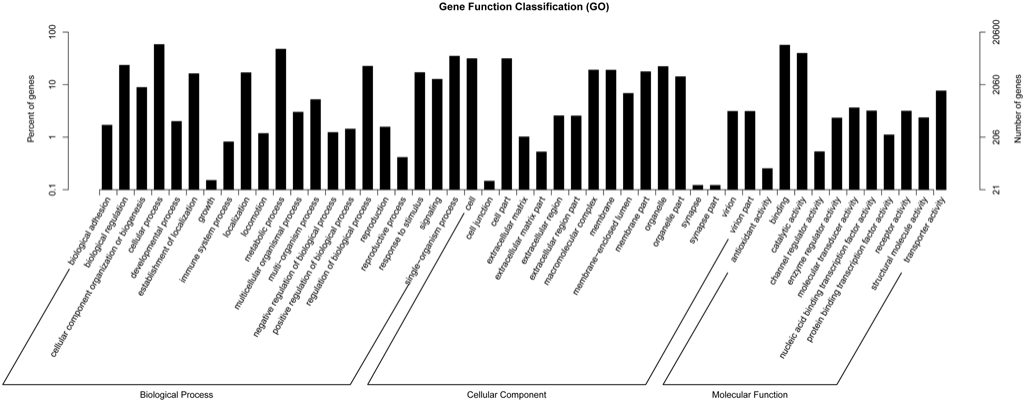
GO (Gene Ontology) categorization (Biological process, Cellular component and Molecular function) of unigenes in the mantle transcriptome of *P*. *yessoensis*. Each annotated sequence is assigned at least one GO term.

According to Gene Ontology (GO), there are 20,600 unigenes assigned to three main functional categories, including biological process, cellular component, and molecular function. Among those annotated unigenes, 48.41% are classified to the category of biological process, 30.55% to cellular component, and 21.04% to molecular function (Fig 4). There are a total of 21 subcategories grouped to the category of biological process. The maximum number of genes (11,955; 21.01%) is involved in the subcategory of cellular process, followed by metabolic process (9,758; 17.15%) and single-organism process (7,171; 12.60%). The category of cellular component, containing 17 subcategories, more genes are involved in the subcategories of cell and cell part, accounting for 17.94% and 17.93%, respectively. For the category of molecular function, most genes are assigned to the subcategories of binding (11,619; 46.99%) and catalytic activity (8,136; 32.90%), while only 52 genes (0.21%) are assigned to the subcategories of antioxidant activity.

**Fig 5:**
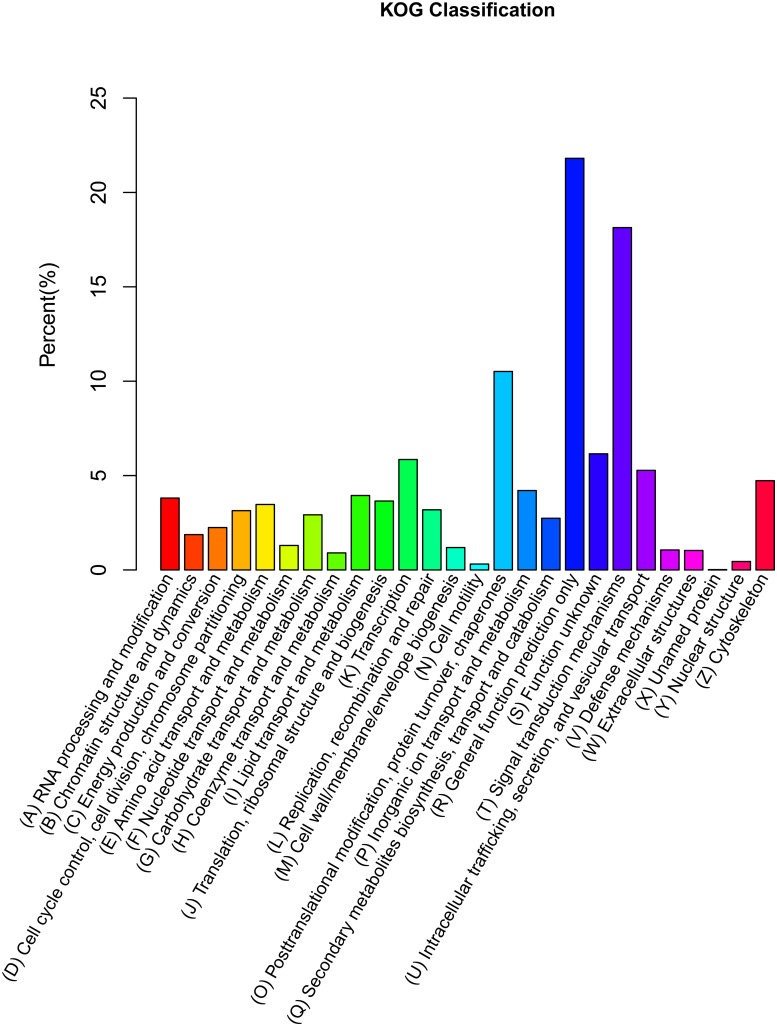
KOG (euKaryotic Ortholog Groups) classification of putative proteins for the mantle transcriptome of *P*. *yessoensis*.

A total of 10,954 annotated genes are assigned to the 26 ortholog groups in KOG database (Fig 5). More than half of the unigenes are distributed in the three main groups, (R) General function prediction only (21.81%), (T) Signal transduction mechanisms (18.14%), and (O) Posttranslational modification, protein turnover, chaperones (10.52%). In contrast, there are less than 10% of the unigenes unevenly distributed in each of the remaining subcategories, such as (Y) Nuclear structure and (N) Cell motility, accounting for 0.45% and 0.31%, respectively.

**Fig 6:**
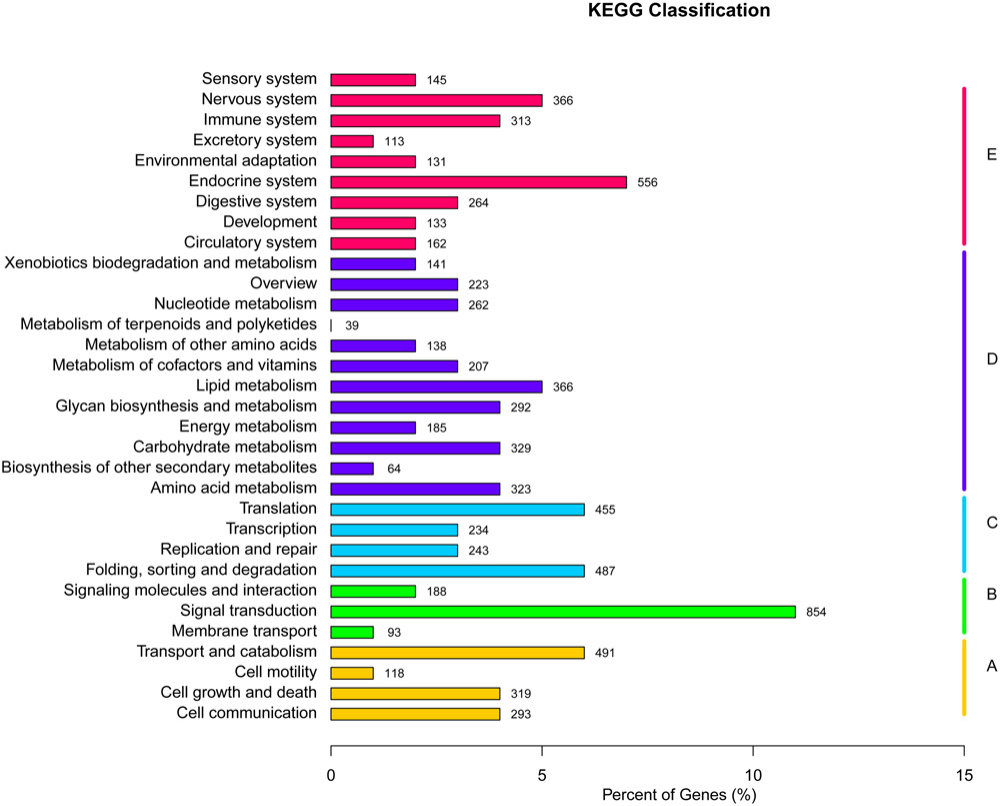
KEGG (Kyoto Encyclopedia of Genes and Genomes) assignment of unigenes in the mantle transcriptome of *P. yessoensis*. A, Cellular Processes; B, Enviromental Information Processing; C, Genetic Information Processing; D, Metabolism; E, Organismal Systems.

The biological pathways for the unigenes were determined by using KEGG database, with 7,989 unigenes assigned to five specific pathways, including metabolism, genetic information processing, environmental information processing, cellular processes, and organismal systems. To sum up, the annotated unigenes in KEGG database are involved in 259 different pathways (Fig 6). More than 50% of unigenes are classified to the two primary pathway groups of metabolism (Fig 6D, 32.16%) and organismal systems (Fig 6E, 27.33%), followed by genetic information processing (Fig 6C, 17.76%), cellular processes (Fig 6A, 15.28%), and environmental information processing (Fig 6B, 14.21%). Among all the KEGG classification, the largest number of unigenes (854) is assigned to the pathway of signal transduction, representing for 75.24% of the total annotated unigenes in the category of environmental information processing, followed by endocrine system pathway (556) in organismal systems category (Fig 6).

Additionally, there are a total of 118 unigenes identified in calcium signaling pathway. Among those, almost one-third (35 annotated unigenes) are homologous with calmodulin and related proteins. Another important pathway of melanogenesis potentially involved in shell pigmentation is identified in the mantle transcriptome of *P*. *yessoensis*, consisting of 86 annotated unigenes, which are mainly homologous to adenylate cyclase 1, protein kinase A, tyrosinase, calmodulin, and guanine nucleotide-binding protein G (data are not shown).

### Screening of Genes Involved in Biomineralization and Melanin Biosynthesis

Alignments of annotated unigenes with the sequences of proteins known to be associated with biomineralization in molluscs reveal 237 unigenes with strong homologies to known proteins (S1 Dataset). As expected, many of the reported genes potentially related to biomineralization, such as calmodulin and calcium-dependent protein, are involved in calcium signaling pathway in *P*. *yessoensis*. The existence of known proteins related to biomineralization, such as bone morphogenetic protein, calmodulin, carbonic anhydrase, chitin synthase, collagen alpha chain, c-type lectin, ferritin, nicotinic acetylcholine receptor subunit, tyrosinase-like protein, leucine-, glutamate- and lysine-rich protein, and some other related proteins are also identified in this study.

To obtain more information about the shell formation process in *P*. *yessoensis*, the prediction of genes potentially related to biomineralization were made in the following steps. The initial step for the prediction of genes was to screen for the proteins with signal peptides using SignalP. As a result, there are a total of 1088 unigenes predicted to have a signal peptide. After the filtering of unigenes by TMHMM, GPI Predictor, and TargetP, a total of 356 unigenes are obtained. The remaining proteins were then screened, using XSTREAM, for tandem repeats, resulting in the identification of 73 unigenes with tandem repeats. Of these 73 unigenes, three have been identified by alignments with known proteins. The remaining 70 unigenes, encoding 67 proteins, are predicted to be potentially related to biomineralization (S1 Dataset).

There are 86 unigenes involved in the melanogenesis pathway in the KEGG database, and 34 unigenes are found in the pathway of tyrosine metabolism. One unigene (comp449846_c0) for putative tyrosinase-like protein tyr-1 is detected in both of these pathways. We then search for genes related to melanogenesis or melanin biosynthesis, using the key word “melanogenesis” and “melanin”, resulting in 88 unigenes homologous to more than 60 known proteins, such as calmodulin, calmodulin-like protein, adenylate cyclase, tyrosinase-like protein, frizzled gene family and so on (S2 Dataset). Of these unigenes, 69.0% are assigned to the T category (Signal transduction mechanisms) by the KOG description (Fig 5).

Finally, eliminating replicates of unigenes identified by different methods, we obtain 121 unigenes, encoding 93 known proteins, which are speculated to be potentially related to melanogenesis or melanin biosynthesis (S2 Dataset). Among these unigenes, many of them (30) are homologous with calmodulin and related proteins. Identified biomineralization genes are compared with those involved in melanogenesis, which uncovers 23 unigenes involved in both processes. These dual functioning unigenes are assigned to eight unique proteins according to their Nr descriptions, including calmodulin, calmodulin-2, calmodulin-like protein, troponin c-long calcium/calmodulin-dependent protein kinase type II, Ca(2+)/calmodulin-responsive adenylate cyclase, putative tyrosinase-like protein tyr-1, and membrane primary amine oxidase (see Table 3). According to the functional description, these unigenes are assigned to four main categories, calmodulin and related proteins (EF-Hand superfamily), Ca2+/calmodulin-dependent protein kinase (EF-Hand protein superfamily), adenylate/guanylate cyclase, tyrosinase family, and primary-amine oxidase.

**Table 3:** The unigenes related to both biomineralization and melanin biosynthesis in the mantle transcriptome of *P*. *yessoensis*.

Unique proteins	Unigene name	FPKM (max.)	Description
Calmodulin	comp99133_c0; comp91863_c0; comp86905_c0; comp65888_c0; comp378491_c0; comp266989_c0; comp187533_c0; comp341061_c0; comp84559_c0; comp60946_c0; comp281906_c0; comp629889_c0; comp221619_c0; comp67649_c1	22.45	Calmodulin and related proteins (EF-Hand superfamily)
Calmodulin-2	comp90730_c0	71.53	Calmodulin and related proteins (EF-Hand superfamily)
Calmodulin-like protein	comp59366_c0	5.71	Calmodulin and related proteins (EF-Hand superfamily)
troponin C-long	comp39291_c0	393.07	Calmodulin and related proteins (EF-Hand superfamily)
Calcium/calmodulin-dependent protein kinase type II	comp98958_c0	19.62	Ca2+/calmodulin-dependent protein kinase (EF-Hand protein superfamily)
Ca(2+)/calmodulin-responsive adenylate cyclase	comp95782_c1	2.53	Adenylate/guanylate cyclase
Putative tyrosinase-like protein tyr-1	comp449846_c0	1.58	Tyrosinase family
Membrane primary amine oxidase	comp20771_c0	2.21	Primary-amine oxidase

## Discussion

Mantle is the tissue in bivalves, which secretes biomineralization proteins as its edge to induce shell formation and pigmentation [5, 31, 32, 49]. For bivalves, the comprehensive description of mantle transcriptome has enabled us to generate a global view of the transcriptome and its organization, and identify a series of important metabolic pathways and genes related to biological functions. Previous transcriptome sequencing of *P*. *yessoensis*, sampling from a variety of developmental stages and adult tissues, produced 970,422 (304 Mb) raw reads, using the 454 GS FLX platform, yielding 805,330 (231 Mb) reads after filtering [39]. Compared to this previously sequenced transcriptome, we find a similar number of unique protein-coding genes by functional annotation (28,228 *vs*. 25,237) but slightly different patterns of functional annotations for the unigenes. For example, more subcategories have been classified within the category of biological process and cellular component in the mantle transcriptome. Several subcategories in the category of biological process, such as locomotion, biological adhesion, single-organism process, establishment of localization, signaling, and immune system process, have been newly classified in the mantle transcriptome. Similarly, subcategories of the category of cellular component, such as membrane part, membrane and virion, have also been newly discovered in the present study. One possible explanation is that the expression level of these related proteins is too low to be detected in the samples derived from mixed tissues in Hou et al.’s study [39]. Another explanation for the difference is that these new groups of genes are actively expressed in the mantle tissue of *P*. *yessoensis*, because they are related to its biological functions. Similar subcategories and proportions of GO annotations were also revealed by the transcriptome analysis of mantle tissue from two pearl oyster species, *Pinctada maxima* and *P*. *martensii*, and one scallop species, *Chlamys farreri* [30, 31, 40]. For instance, most unigenes were assigned to the subcategories of binding and cellular process in the category of molecular function and biological process, respectively, as indicated in the assembled mantle tissue transcripts from above species. We then tested the number and proportions of GO annotations from the present study against those from previous studies, using the chi-square goodness-of-fit test, indicating the consistency of GO annotation results among the mantle transcriptomes of the bivalve species (*P* > 0.05). Their high or specific expression in mantle was proved by comparing gene expression level (RPKM) with that in other organs in the Pacific oyster [24]. These evidences further imply that some of mantle-specific or highly expressed genes in bivalves may be involved in shell formation, and others may be related to the general function of the mantle, such as signal transduction, locomotion, and immune, and so on. Additionally, 69% of the screened unigenes related to melanin biosynthesis are assigned to the KOG class of (T) Signal transduction mechanisms, as shown in Fig 5. It is suggested that melanin biosynthesis were likely to be involved in signal transduction, and melanogenesis-related genes are most likely sorted into this category. Those defined terms representing gene product properties will provide a valuable gene resource for functional genomic studies.

The hard shell of molluscs, which is secreted by the mantle, serves not only for muscle attachment, but also for protection from predators and mechanical damage. Shell formation, results from calcium carbonate deposition, which has attracted substantial research and a number of important genes involved in shell biogenesis have been identified and characterized in the bivalve species, *P*. *maxima*, *P*. *martensii*, and *C*. *farreri*, by studying the transcriptome of the mantle [30, 31, 40]. In the present study, 102 biomineralization-related genes are identified in the mantle transcriptome of *P*. *yessoensis*, comparable to that of *C*. *farreri* (96) and two times more than that of *P*. *martensii* (49). Mollusc shell formation is highly controlled by calcium metabolism process, and the transportation and metabolism of calcium ions make a substantial contribution to the biomineralization process [10]. Therefore, the unigenes found in calcium signaling pathway could potentially play an important role in shell formation of *P*. *yessoensis*. In this study, alignments between unigenes and reported proteins related to biomineralization reveal a large number of potential genes involved in calcium metabolism in the mantle transcriptome of *P*. *yessoensis*. For example, calmodulin and calmodulin-like protein are highly expressed in *P*. *yessoensis* mantle, with remarkably high values of the fragments per kilobase per million fragments (FPKM; S1 Dataset), which is consistent with the previous studies indicating that calmodulin and calmodulin-like protein are two impor`tant proteins related to biomineralization in pearl oysters and scallops [30, 31, 50, 51]. In the pearl oyster, calmodulin-like protein induces the nucleation of aragonite, through binding with the 16-kDa protein, and regulates the growth of calcite in the prismatic layer, while calmodulin may regulate calcium transport and secretion [50]. In comparison with pearl oysters, the Yesso scallop has a relatively smaller proportion of aragonite in its shell and lacks the 16-kDa protein in the mantle, suggesting that the function of calmodulin-like protein in the scallops *C*. *farreri* and *P*. *yessoensis* may be different from that in pearl oysters during the process of calcium metabolism [30].

According to the previous reports, carbonic anhydrase and alkaline phosphatase could catalyze HCO_3_^-^ formation and facilitate calcium carbonate crystal formation in the nacreous layer of the pearl oyster [52, 53]. However, relatively low FPKM values of carbonic anhydrase and alkaline phosphatase are detected in the mantle of *P*. *yessoensis* (S1 Dataset). In contrast, alkaline phosphatase is moderately expressed in the *C*. *farreri* mantle, with FPKM values of 91.9 [30]. The quite abundantly expressed genes in the *P*. *yessoensis* mantle, with the maximum number of 41 unigenes homologous to different types of collagen genes, has been hypothesized to be involved in the nucleation of organized calcium carbonate deposition [49]. However, no collagen gene was reported in the mantle transcriptomes of *P*. *maxima*, *P*. *martensii*, and *Chlamys farreri* [30, 31, 40]. Perlucin, homologous with the same protein in *C*. *gigas*, was only identified in the mantle trancriptome of the scallop *P*. *yessoensis* and *C*. *farreri* but was not reported in that of the pearl oysters, which are able to nucleate calcium carbonate layers on calcite surfaces [16, 30, 54]. Additionally, there are some other calcium-binding proteins such as sarcoplasmic calcium-binding protein and EF-hand calcium-binding protein, also identified in the mantle of *C*. *farreri* and *P*. *martensii*, which might play important roles in calcium metabolism formation by regulating calcium uptake and transport [55, 56].

In this study, unigenes homologous to other prot**e**ins related to biomineralization in molluscs, such as chitin synthase, heat shock protein 70, sodium/calcium exchanger, and tyrosinase-like protein, are also confirmed in the mantle transcriptome of *P*. *yessoensis*. Chitin synthase, which is not only identified in *P*. *yessoensis* but also in *C*. *farreri* and is responsible for chitin deposition, is thought to play a functional role in the cytoskeletal forces of the precisely controlled mineral deposition process in molluscs [14, 30, 57]. For molluscs, the shell has a composite structure of calcium carbonate crystals in the polymorph of calcite and aragonite, which are potentially regulated by shell matrix proteins in the crystallization process of calcium carbonate. Some of matrix proteins, such as MSP-1 and tyrosinase-like protein, are probably related to the formation of calcite, whereas other matrix proteins such as Pif and PfN23 have been identified to regulate the formation of aragonite crystals in molluscs [30, 58, 59]. In the present study, four matrix proteins, including perlucin, tyrosinase-like protein 1, tyrosinase-like protein 3 and N151, were identified and annotated from 31 unigenes. As reported, tyrosinase-like protein 1 and 3 are potentially participated in the formation of calcite shell layers, while perlucin may be related to aragonite formation in *P*. *yessoensis* [30]. In contrast, N151 was not characterized to be involved in calcite or aragonite. Despite this, the function of these matrix proteins in the crystallization process of calcium carbonate remains largely unknown in *P*. *yessoensis*. The nacre proteins, such as Nacrein, N16, and N19, which are membrane protein-like components specific to the nacreous layer that generate aragonite crystals in pearl oyster shells, are not found in the mantle transcriptomes of *P*. *yessoensis* and *C*. *farreri* [30, 31, 52, 60, 61]. The absence of these nacre proteins in scallops is likely the result of different shell structures in scallops and pearl oysters. For example, the shells of scallops are reported to be mainly composed of calcite, with only a small proportion of the shell composed of aragonite; on the other hand, a large proportion of aragonite was detected in the shells of pearl oyster [30, 31, 62].

The visible pigmentation of the skin, hair and eyes of mammals and other vertebrates results mainly from the presence and distribution of melanin pigments. The pigment melanin is produced by melanocytes in melanosomes during a complex process of melanogenesis [63]. For vertebrates, melanin biosynthesis starts with the hydroxylation of L-tyrosine and further oxidation to dopaquinone, which is catalysed by the rate-limiting enzyme of tyrosinase [64]. In contrast, little is known for the mechanism of melanogenesis in invertebrates, except for the insects and cuttlefish [65, 66]. Despite the functional differences, the knowledge of vertebrate pigmentation could shed lights on the understanding of melanin biosynthesis in invertebrates, including marine bivalves. For *P*. *yessoensis*, the pathways of tyrosine metabolism and melanogenesis detected in the mantle transcriptome might play fundamental roles in the biology of shell pigmentation, and will provide crucial insights into the regulation of melanin biosynthesis. Numerous genes involved in those two pathways are characterized in the mantle transcriptome of *P*. *yessoensis*, such as adenylate cyclase type 1, calmodulin-like protein, calmodulin, G-protein alpha subunit, glycogen synthase kinase-3, and tyrosinase-like protein, monoamine oxidase A, and tyrosine hydroxylase, which are potentially responsible for the brown pigmentation of *P*. *yessoensis* in the right valve (Fig 1). Furthermore, the dual functioning proteins in both biomineralization and melanogenesis such as calmodulin and related proteins, Ca2+/calmodulin-dependent protein kinase, and adenylate/guanylate cyclase, are all proved to play important roles in calcium metabolism, suggesting that they are not only make a substantial contribution to the biomineralization process by regulating calcium uptake, transport and secretion, but also presumably play important roles in shell pigmentation in this species. Additionally, another notable protein, tyrosinase-like protein, seems to play the similar roles with the above proteins, suggesting that the functions of tyrosinase-like protein in *P*. *yessoensis* are probably not limited to shell formation but are also involved in the pigmentation on the shells, which was also evidenced in *C*. *gigas* [24]. As mentioned, tyrosinase is a key enzyme in melanogenesis, and the localization of tyrosinases in pigmented regions has been reported in various organisms [19, 67, 68]. It is therefore suggested that *P*. *yessoensis* tyrosinases regulated by tyrosinase-like protein are probably secreted from the mantle and transported to the calcified prismatic shell layer, where they contribute to melanin biosynthesis. Notably, the link between calcium metabolism and melanin biosynthesis was previously examined in vertebrate animals. As reported in the barn owl, eumelanin integuments such as spotted feather parts are enriched with calcium, which suggests that calcium is a component of the pigments responsible for the production of black spots [69]. Additionally, it is further confirmed that melanin-based coloration is associated with a number of physiological processes requiring calcium in this bird species [70]. Our findings revealed from the transcriptomic data support the idea that there might be a link between melanin biosynthesis and calcium metabolism, and further provide new evidence on gene expression for the understanding of pigmentation and biomineralization not only in invertebrates, but also probably in vertebrates.

## Conclusions

In conclusion, we report the first comprehensive transcript dataset of the mantle transcriptome of *P*. *yessoensis*. The identified and annotated transcripts will provide valuable genomic resources for the understanding of its unique biological characteristics, such as shell structures and pigmentation in this species. The significant number of genes is characterized to be associated with both biomineralization and melanogenesis, mainly homologous to calmodulin and related proteins, adenylate/guanylate cyclase, and tyrosinase-like protein. It is speculated that these genes not only make a substantial contribution to the process of shell formation, but also play important roles in shell pigmentation in molluscs, suggesting that there might be a link between calcium metabolism and melanin biosynthesis in invertebrates. These findings could enrich the theoretical basis of biomineralization process, assist our understanding of the sophisticated processes of shell formation and pigmentation in this species and other bivalves, and also provide new insight into the biology of pigmentation and biomineralization in vertebrate and invertebrates.

## Supporting Information

S1 Dataset Alignment results with genes potentially related to biomineralization in the mantle transcriptome of *P*. *yessoensis*. (XLSX)

S2 Dataset Alignment results with genes potentially related to melanogenesis in the mantle transcriptome of *P*. *yessoensis*. (XLSX)
